# U-shaped association between social media usage frequency and suggestibility by internet health information in Chinese online population with pre-diabetes and diabetes: a cross-sectional study

**DOI:** 10.1186/s12889-025-22724-1

**Published:** 2025-04-24

**Authors:** Mutong Chen, Xiaobing Lin, Rui Zhou, Guanhua Fan

**Affiliations:** 1 Health Management Center of Outpatient Department, Cancer Hospital of Shantou University Medical College, No. 7 Raoping Road, Shantou, 515031 China; 2 Shantou University Medical College, Shantou, China; 3 Guangzhou Red Cross Hospital, Guangzhou, China

**Keywords:** Diabetes Mellitus, Self-Management, Social Media, Health Behavior, Internet

## Abstract

**Background:**

Internet-based self-management of diabetes has been demonstrated to be effective. The frequency of social media is believed to be associated with diabetes management, yet the quality and applicability of online information are still subject to debate. The dynamic nature of online information complicates its study, making it crucial to further assess the online behaviors and psychological aspects of the population engaged in online blood glucose management.

**Objective:**

The objective of this study was to explore the relationship between the frequency of social media usage and the suggestibility of internet health information.

**Methods:**

This study is a secondary analysis based on data obtained from a prior cross-sectional survey conducted in multiple online diabetes communities in China, which received a total of 5,504 responses, ultimately including 1,062 individuals with diabetes or prediabetes for analysis. Frequency of social media usage was measured using a 6-point Likert scale across five items, evaluating the frequency of use on platforms such as WeChat, Weibo, QQ, TikTok, and others. Suggestibility by internet health Information was assessed using a 5-point Likert scale across nine items reflecting individuals’ trust in, discussion of, and engagement with internet health information and communities. Data analysis was conducted using R language and EmpowerStats software, encompassing Chi-square tests, U tests, multifactor linear regression analysis, smooth curve fitting, and subgroup and interaction effect analysis.

**Results:**

After adjusting for other factors, there was a positive correlation between the frequency of social media and the suggestibility of internet health information cues (β = 0.27, 95% *CI*: 0.24 to 0.30, *P* < .001). A nonlinear relationship was identified, with a turning point at 3.8. When the social media usage score is below 3.8, each unit increase in the social media score corresponds to a decrease of 0.12 in the internet health trust score (95% *CI*: -0.19 to -0.05, *P* < .001). Conversely, when the application usage score is 3.8 or above, each unit increase leads to an increase of 0.46 in the internet health suggestibility score (95% *CI*: 0.42 to 0.50, *P* < .001). Interaction analysis revealed significant interactive factors affecting the relationship between social media usage frequency and suggestibility by internet health information, including gender (*P* = .045), age (*P* < .001), body measurement index (*P* = .03), sleep latency (*P* = .001), self-monitoring of blood glucose frequency (*P* = .001), and glycemic control status (*P* < .001).

**Conclusions:**

Once a certain threshold of social media usage frequency is reached, suggestibility by internet health information cues increases with increased usage. Monitoring social media usage frequency could thus provide insightful indicators for evaluating the online behavioral and psychological profiles of individuals engaged in online blood glucose management.

## Introduction

As a complex chronic disease, diabetes necessitates effective management through self-care knowledge and practices [1]. In the management of diabetes, patients’ understanding of their own condition and self-care are particularly crucial [2, 3]. Self-management—encompassing both maintaining healthy behaviors and appropriate medication usage—can significantly delay the progression of diabetes and diminish the risk of associated complications [4–7]. However, self-management of diabetes is a complex and arduous process, requiring patients to make numerous daily decisions such as meal planning, medication timing, and exercise schedules [3]. In practice, effective diabetes management extends beyond regular blood glucose monitoring and medication adherence, it also involves meticulous control over diet and sustained physical activity [8]. This underscores the importance of continuous behavioral support in enhancing the efficacy of diabetes management protocols. The advent of information technology and the proliferation of the internet have revolutionized this aspect, particularly through diabetes management applications that streamline self-care processes [9]. With the rapid advancement of internet technologies and the widespread availability of smartphones, social media have emerged as powerful tools for diabetic patients to access medical guidance and manage their condition effectively [9].

The internet has undeniably enriched diabetes intervention strategies, especially in facilitating self-management [10]. Internet-based diabetes interventions have shown significant positive outcomes, as evidenced by the reduction in glycosylated hemoglobin levels, weight changes, improvements in psychological well-being, and enhanced quality of life [11–13]. Social media serve as critical components of the internet-based chronic disease management framework, with health information dissemination via social media-related applications proving effective [14]. Such interventions are deemed beneficial for the self-management endeavors of diabetic individuals [15]. Moreover, prior research has indicated a preference among users for interactive health information, who are more willing to access health-related information through social media rather than via traditional search [16]. Therefore, studying how the frequency of social media use influences diabetes patients’ acquisition, processing, and application of health information can provide important insights into the behavioral patterns of online blood glucose management communities.

With the rapid advancement of technology, the risk of patients encountering inaccurate or misleading information has significantly increased [17], and the applicability of different intervention guidelines varies among individuals [18]. Consequently, the credibility and applicability of online information require further scrutiny [19]. n recent years, a growing body of research has focused on the quality of internet health information, including investigations into the quality of diabetes-related health content [20]. These studies have further highlighted issues such as the poor quality and incompleteness of internet health information [21–24]. However, due to the dynamic and variable nature of online content, it remains challenging to draw definitive conclusions about its quality [25]. Within this complex context, patients’ trust in internet health information and their ability to internalize it have become a critical factor influencing the effectiveness of information acquisition and application. Therefore, there is a pressing need for more robust methods to assess the behaviors and psychological aspects of online glucose management populations in obtaining internet health information amidst dynamic changes.

Overall, there are numerous barriers in the process from obtaining information online to its effective application in diabetes self-management. Objectively, the quality and applicability of online information require further validation. Subjectively, on one hand, patients need the ability to discern the reliability of online information [26]; on the other hand, the susceptibility of patients to the suggestiveness of internet health information, from a more individualized perspective, also warrants attention.

Suggestibility, as a personality trait, reflects an individual’s tendency to accept and internalize information, thereby influencing their behavior [27, 28]. Individuals with higher suggestibility are more likely to be influenced by online health information, leading to behavioral adjustments, active participation in online community activities, and dissemination of the information they acquire. At the same time, social media usage frequency, as a measurable behavioral indicator, may reflect patients’ patterns of exposure to and internalization of health information to a certain degree. In Chapoton’s survey study, individuals with higher suggestibility tended to have more social connections on Facebook, which indirectly influenced alcohol abuse [27]. Similarly, a prior survey conducted in China involving 463 internet users found that frequent social media users exhibited better health outcomes, with improvements in health behaviors mediated by trust in health knowledge [29]. While the potential association between suggestibility by internet health information and social media usage has not been fully explored, studying this independent association from a cross-sectional perspective holds significant importance. On the one hand, such research can help uncover the psychological mechanisms underlying the internalization of health information in a dynamic online environment. On the other hand, it can provide theoretical insights for standardizing internet health information dissemination, improving diabetes education, and optimizing blood glucose management strategies. Moreover, directly assessing patients’ suggestibility in social contexts poses certain challenges, whereas monitoring social media usage frequency offers a simpler and more feasible alternative.

In this study, we aim to provide a new perspective on understanding online health behavior patterns and offer practical guidance for designing internet-based health education and behavioral interventions by exploring the independent association between social media usage frequency and suggestibility toward internet health information. This exploratory research is based on a secondary analysis of survey data previously collected from internet users in China with pre-diabetes and diabetes.

## Methods

### Participants and data collection

This investigation constitutes a secondary analysis of a prior cross-sectional survey that we conducted [30]. It was conducted in accordance with the ethical principles outlined in the Declaration of Helsinki and was approved by Ethics Committee of Shantou University Medical College(Code: SUMC-2021–064). Written informed consent was obtained from all participants prior to their inclusion in the study. The initial survey was executed over the period from March 15 to May 15, 2022. Distribution of the survey questionnaires occurred across multiple platforms: glucose management forums (e.g., “Sweet Home,” “Glucose Column,” “Hyperglycemia Column,” “Diabetes Column”), QQ groups (“Diabetes Exchange Group”), and WeChat groups focused on glucose management (“Glucose Friends Support Exchange Group”).

**Fig. 1:**
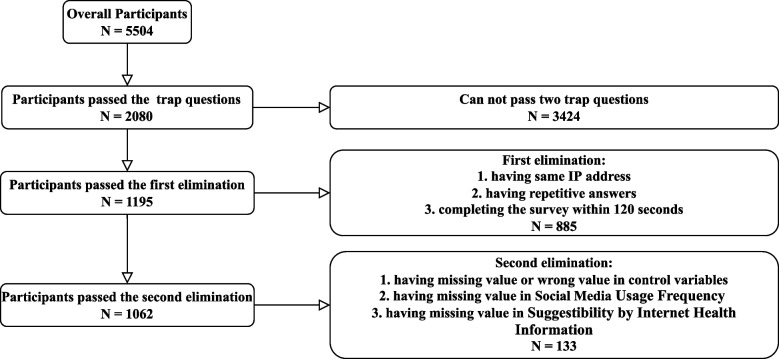
Flow diagram of sample selection

The inclusion criteria were delineated as follows: 1) Subjects exhibiting glucose abnormalities, which included individuals with diabetes (fasting glucose ≥ 7.0 mmol/L, postprandial glucose ≥ 11.1 mmol/L), prediabetes (fasting glucose 6.1–6.9 mmol/L, postprandial glucose < 7.8 mmol/L), impaired fasting glucose (fasting glucose 5.6–6.1 mmol/L, postprandial glucose < 7.8 mmol/L), or impaired glucose tolerance (fasting glucose < 7.0 mmol/L, postprandial glucose 7.8–10.9 mmol/L), all of whom consistently utilized glucose management applications. 2) Normoglycemic participants who engaged with individuals with glucose abnormalities, partook in online information exchanges, utilized glucose management applications, and expressed a desire for related assistance and support. 3) Participants capable of completing the questionnaire independently or with aid. 4) Participants without any history of psychiatric disorders or cognitive impairments. The exclusion criteria were specified as follows: 1) Any two or more survey samples sharing the same network IP address; 2) Samples characterized by a high degree of answer repetitiveness; 3) Samples completed in less than 120 s; 4) Responses that were inconsistent, illogical, or incomplete; and 5) Samples missing essential variables required for this study. Ultimately, 1062 valid questionnaires were included in all subsequent analyses. The processes of inclusion and exclusion are illustrated in Fig. 1.

### Variable

#### Predictive variable

The frequency of social media usage was evaluated based on five items on a 6-point Likert scale (1 = “never”, 2 = “less than once a month”, 3 = “once a month”, 4 = “several times a month”, 5 = “several times a week”, 6 = “every day”), covering the following platforms: (1) WeChat (including WeChat groups), (2) Weibo, (3) QQ (including QQ groups), (4) Tik Tok, (5) others.

#### Outcome variable

The suggestibility by internet health Information was assessed using a 5-point Likert scale (1 = “strongly disagree”, 2 = “disagree”, 3 = “neutral”, 4 = “agree”, 5 = “strongly agree”) across nine items, which include: 1) Internet health information is an important reference for my medical or health decisions; 2) I discuss health information found online with my doctor; 3) I discuss internet health information with family or friends; 4) Doctors may affirm the outcomes obtained from my interactions with group members; 5) I have encountered group members who frequently share knowledge and experiences; 6) Although I do not directly know some members of online groups, I trust them; 7) I often discuss healthy lifestyles with my friends offline; 8) I engage in online discussions about various health lifestyle topics; 9) I am increasingly participating in health management themed groups or platforms.

The suggestibility by internet health Information scale was developed in Chinese, based on insights from social media literature and real-world scenarios relevant to Chinese online users managing blood glucose. Most items were originally designed in Chinese [31], while a small portion was adapted from English sources [16, 27]. For these adapted items, a back-translation procedure was conducted: two bilingual experts independently translated the items into Chinese and back into English, with discrepancies resolved through expert discussions to ensure cultural and linguistic equivalence. To enhance content validity and clarity, experts in psychology and diabetes management reviewed the items during the design phase. Before the normal distribution of our questionnaire, a pilot test involving 50 participants from the target population was conducted to evaluate reliability and validity. The scale demonstrated good internal consistency (Cronbach’s α = 0.85), and exploratory factor analysis revealed a single-factor structure with factor loadings ranging from 0.65 to 0.82, indicating satisfactory construct validity. We also conducted reliability and validity testing on the data used for the final analysis of current study. The overall sampling adequacy value of the Kaiser–Meyer–Olkin test is 0.92, indicating that the data is highly suitable for factor analysis. The measure of sampling adequacy for each item is ranging from 0.91–0.93, further supporting the appropriateness of the data. The Cronbach’s α coefficient is 0.88, demonstrating good internal consistency of the scale.

#### Control variables

**Fig. 2:**
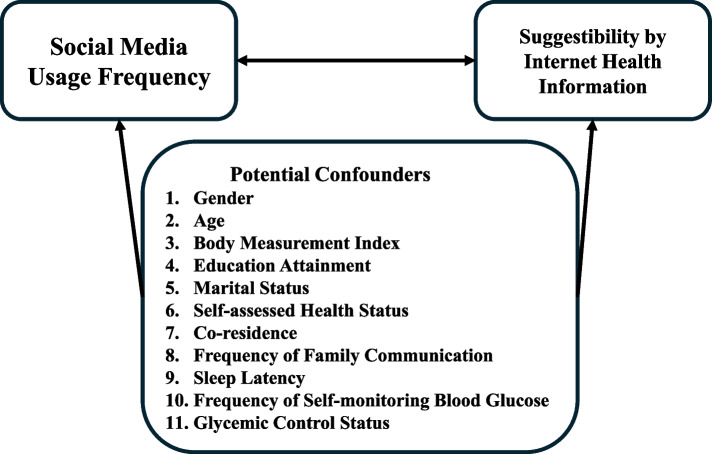
Diagram of the conceptual framework

We selected variables with potential confounding effects for control based on relevant literature and logical inference from the variables collected in the survey questionnaire.including gender [32], age (categorized as ≤ 20, 21–40, ≥ 41) [33], Body measurement index (BMI) divided into four categories based on thresholds of 18.5, 24, and 28, educational attainment (Middle school or below, Junior college, College and above) [34], marital status [35], self-assessed health status (categorized as Having any disease, Insufficient energy, Normal health), co-residence(yes = not living alone)), frequency of family communication (Several days, Every day, Always), sleep latency (Within 15 min, 16–30 min, 31–60 min, ≥ 61 min) [36], frequency of self-monitoring blood glucose (Never, Occasional, Daily) [37], and the glycemic control status (The proportion of people with poor blood glucose control in the corresponding group was calculated with the threshold of 6.5% glycosylated hemoglobin). Figure 2 demonstrates our conceptual framework when constructing regression models.

### Statistical analysis

Continuous variables, such as social media usage frequency and suggestibility by internet health information, were described using mean ± standard deviation. The Chi-square test was used for categorical variables, whereas the Mann–Whitney U test was applied for continuous variables, with results considered significant at *P* < 0.05. Multifactor linear regression was employed to adjust for confounders. Relationships between social media usage frequency and suggestibility by internet health information were explored using generalized additive models and smoothing curves. The robustness of the findings was validated through stratified analysis. Data analysis was facilitated by the R package (version 4.1.3, http://www.Rproject.org) and EmpowerStats software (http://www.empowerstats.com).

## Results

### Descriptive analysis of selected participants

The study encompassed data from 1062 participants, comprising 405 females (38.1%) and 657 males (61.9%), as shown in Table 1. Age distribution was as follows: 49 participants were under 20 years (4.6%), 840 were between 21 and 40 years (79.1%), and 173 were over 41 years (16.3%). Sixty percent (645 participants) had a BMI within the normal range. The mean scores for social media usage frequency and suggestibility by internet health information were 4.56 ± 0.92 and 4.08 ± 0.55, respectively. Based on the median score of suggestibility, participants were divided into two groups. Significant differences between groups with high and low suggestibility were observed in several variables including gender, age, BMI, education, marital status, health status, family communication frequency, sleep latency, and frequency of glucose self-monitoring, all showing statistical significance. Conversely, no significant differences were noted concerning the adequacy of blood sugar control between the two groups.

**Table 1:** Baseline characteristics of analyzed samples according to suggestibility by internet health information

Suggestibility by IHI	Overall	Low	High	***P***-value
**N**	1062	556	506	
**Gender = female (%)**	405 (38.1)	193 (34.7)	212 (41.9)	.02
**Age group (%)**				**.001**
≤ 20	49 (4.6)	37 (6.7)	12 (2.4)	
21–40	840 (79.1)	442 (79.5)	398 (78.7)	
≥ 41	173 (16.3)	77 (13.8)	96 (19.0)	
**BMI groups (%)**				.046
< 18.5	122 (11.5)	63 (11.3)	59 (11.7)	
[18.5,24)	645 (60.7)	320 (57.6)	325 (64.2)	
[24,28)	167 (15.7)	93 (16.7)	74 (14.6)	
≥ 28	128 (12.1)	80 (14.4)	48 (9.5)	
**Education levels (%)**				.03
Middle school or below	228 (21.5)	120 (21.6)	108 (21.3)	
Junior college	245 (23.1)	146 (26.3)	99 (19.6)	
College and above	589 (55.5)	290 (52.2)	299 (59.1)	
**Marital Status = married (%)**	701 (66.0)	331 (59.5)	370 (73.1)	** < .001**
**Co-residence = Yes (%)**	858 (80.8)	438 (78.8)	420 (83.0)	.10
**Self-report health status (%)**				** < .001**
Having any disease	6 (0.6)	3 (0.5)	3 (0.6)	
Insufficient energy	589 (55.5)	395 (71.0)	194 (38.3)	
Normal health	467 (44.0)	158 (28.4)	309 (61.1)	
**Frequency of family communication (%)**				** < .001**
Several days	228 (21.5)	159 (28.6)	69 (13.6)	
Every day	629 (59.2)	340 (61.2)	289 (57.1)	
Always	205 (19.3)	57 (10.3)	148 (29.2)	
**Sleep Latency (%)**				.04
Within 15 min	203 (19.1)	92 (16.5)	111 (21.9)	
16–30 min	528 (49.7)	295 (53.1)	233 (46.0)	
31–60 min	264 (24.9)	139 (25.0)	125 (24.7)	
≥ 61 min	67 (6.3)	30 (5.4)	37 (7.3)	
**SMBG Frequency (%)**				** < .001**
Never	46 (4.3)	33 (5.9)	13 (2.6)	
Occasional	604 (56.9)	342 (61.5)	262 (51.8)	
Daily	412 (38.8)	181 (32.6)	231 (45.7)	
**GC status = uncontrolled (%)**	390 (36.7)	214 (38.5)	176 (34.8)	.24
**Social media usage frequency (mean (SD))**	4.56 (0.92)	4.16 (0.80)	5.00 (0.84)	** < .001**
**Suggestibility by IHI (mean (SD))**	4.08 (0.55)	3.65 (0.36)	4.54 (0.26)	** < .001**

Data are presented as mean ± SD or *N* (%). The Chi-square test for categorical variables and Mann–Whitney U test for continuous variables were utilized to assess the difference among groups with high and low suggestibility by IHI

*Abbreviations*: *BMI* Body measurement index, *SMBG* Self-monitoring of blood glucose, *GC* Glycemic control, *IHI* Internet health information. Bold *P* values are significant at *P* < .05

### Multivariable linear regression analysis

In this research, multivariable linear regression was utilized to explore the association between the frequency of app usage and the level of suggestibility by internet health information. Analysis was conducted using three distinct models: crude and adjusted (Table 2). The crude model (Model 1) indicated a positive correlation between social media usage frequency and internet health information suggestibility (β = 0.32, 95% CI: 0.29 to 0.35, *P* < 0.001). Model 2, which adjusted for variables such as gender, age group, education levels, marital status, co-residence, family communication frequency, and self-reported health status, yielded similar outcomes (β = 0.26, 95% CI: 0.233 to 0.29, *P* < 0.001). Furthermore, Model 3, which also adjusted for categorical BMI, sleep latency, SMBG frequency, and glycemic control status, maintained this correlation (β = 0.27, 95% CI: 0.24 to 0.30, *P* < 0.001).

**Table 2:** Association between social media usage frequency and internet health information suggestibility among Chinese Online population with pre-diabetes and diabetes, analyzed via linear regression model

	Beta (95% CI)	***P***—value
Model 1	0.32 (0.29, 0.35)	** < .001**
Model 2	0.26 (0.23, 0.29)	** < .001**
Model 3	0.27 (0.24, 0.30)	** < .001**

Model 1 was the crude model; Model 2 was adjusted for gender, age group, education levels, marital status, co-residence, frequency of family communication, and self-report health status; Model 3 included further adjustments for BMI (Categorical), sleep latency, SMBG frequency, and GC status

*Abbreviations*: *BMI* Body measurement index, *SMBG* Self-monitoring of blood glucose, *GC* Glycemic control, *IHI* Internet health information. Bold* P* values are significant at *P* < .05

### Detection of nonlinear association

**Fig. 3:**
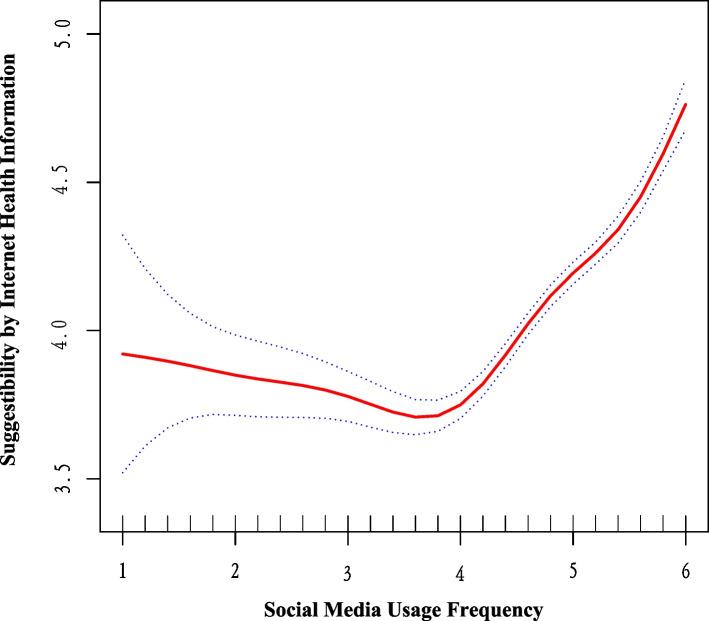
Graphics of smooth curve fittings between the social media usage frequency and suggestibility by internet health information in Chinese online population with pre-diabetes and diabetes. The model was adjusted for gender, age group, education levels, marital status, co-residence, frequency of family communication, BMI(Categorical), self-report health status, sleep latency, SMBG frequency, GC status. Blue bands represent the 95% CI from the fit. The solid red line represents the smooth curve fit between variables. Abbreviations: BMI, body measurement index; SMBG, self-monitoring of blood glucose; GC glycemic control; IHI, internet health information

Generalized additive models and smoothing curves were applied to the analysis. As seen in Table 3, the inflection point K was determined to be 3.8, with the log-likelihood ratio’s *P*-value being less than 0.001, thus a two-segment linear fit was employed to interpret the results. The curve relationship between social media usage frequency and suggestibility by internet health information is displayed in Fig. 3 (Adjusted for gender, age group, education levels, marital status, co-residence, frequency of family communication, BMI (Categorical), self-report health status, sleep latency, SMBG frequency, GC status). When the social media usage frequency score is less than 3.8, there is a negative correlation with the suggestibility of internet health information, where each unit increase in social media usage frequency score results in a decrease of 0.12 in the suggestibility by IHI score (95% CI: -0.19 to -0.05, *P* < 0.001). Conversely, when the social media usage frequency score is equal to or greater than 3.8, there is a positive correlation, with each unit increase in social media usage frequency score resulting in an increase of 0.46 in the suggestibility score (95% CI: 0.42 to 0.50, *P* < 0.001).

**Table 3:** Threshold effect analysis of Social media usage frequency on suggestibility by internet health information in Chinese online population with pre-diabetes and diabetes

Outcome	Adjusted Beta (95% CI), ***P***-value
Fitting by the standard linear model	**0.27 (0.24, 0.30) < .001**
Fitting by the two-piecewise linear	
Inflection point (K)	3.8
Social media usage frequency < 3.8	**-0.12 (-0.19, -0.05) < .001**
Social media usage frequency ≥ 3.8	**0.46 (0.42, 0.50) < .001**
*P* value for log-likelihood ratio	** < .001**

Adjusted for gender, age group, education levels, marital status, co-residence, frequency of family communication, BMI(Categorical), self-report health status, sleep latency, SMBG frequency, GC status

*Abbreviations*: *BMI* Body measurement index, *SMBG* Self-monitoring of blood glucose, *GC* Glycemic control, *IHI* Internet health information. Bold *P* values are significant at *P* < .05

### Interaction and stratification analysis

Stratified analysis was applied to investigate the relationship between social media usage frequency and the suggestibility of internet health information (Table 4). The association between social media usage frequency and suggestibility of internet health information showed no statistically significant differences across various levels of education (*P* = 0.17), marital status (*P* = 0.73), living conditions (*P* = 0.81), frequency of communication with family (*P* = 0.12), and self-assessment of health (*P* = 0.28). Conversely, significant results were observed in the interaction analysis for SMBG frequency (*P* = 0.001) and GC status (*P* < 0.001).

**Table 4:** Interaction and stratification analysis for the association between social media usage frequency on suggestibility by internet health information in Chinese online population with pre-diabetes and diabetes

Stratification variables	Adjusted Beta (95% CI)	Interaction ***P*** value
**Gender**		.045
male	0.29 (0.25, 0.33)	
female	0.23 (0.19, 0.28)	
**Age group**		** < .001**
≤ 20	0.43 (0.33, 0.54)	
21–40	0.27 (0.24, 0.31)	
≥ 41	0.17 (0.11, 0.24)	
**Education levels (%)**		.17
Middle school or below	0.31 (0.25, 0.37)	
Junior college	0.27 (0.22, 0.33)	
College and above	0.24 (0.21, 0.28)	
**Marital Status**		.73
Others	0.27 (0.23, 0.32)	
Married	0.26 (0.22, 0.30)	
**Co-residence**		.81
Yes	0.26 (0.23, 0.30)	
No	0.27 (0.21, 0.34)	
**Frequency of family communication**		.12
Several days	0.32 (0.26, 0.38)	
Every day	0.25 (0.21, 0.29)	
Always	0.24 (0.18, 0.31)	
**BMI groups (%)**		**.03**
< 18.5	0.25 (0.18, 0.33)	
[18.5,24)	0.25 (0.21, 0.28)	
[24,28)	0.27 (0.20, 0.34)	
≥ 28	0.39 (0.30, 0.47)	
**Self-report health status**		.28
Having any disease	-0.02 (-0.38, 0.34)	
Insufficient energy	0.27 (0.23, 0.31)	
Normal health	0.26 (0.22, 0.31)	
**Sleep Latency**		**.001**
Within 15 min	0.17 (0.12, 0.23)	
16–30 min	0.31 (0.26, 0.35)	
31–60 min	0.31 (0.25, 0.37)	
≥ 61 min	0.22 (0.12, 0.32)	
**SMBG Frequency**		**.001**
Never	0.17 (0.07, 0.28)	
Occasional	0.24 (0.20, 0.28)	
Daily	0.34 (0.29, 0.39)	
**GC status**		** < .001**
controlled	0.23 (0.20, 0.26)	
uncontrolled	0.36 (0.31, 0.42)	

Each stratification was adjusted for gender, age group, education levels, marital status, co-residence, frequency of family communication, BMI(Categorical), self-report health status, sleep latency, SMBG frequency, GC status, except the stratification factor itself. *Abbreviations*: *BMI* Body measurement index, *SMBG* Self-monitoring of blood glucose, *GC* Glycemic control, *IHI* Internet health information. Bold *P* values are significant at *P* < .05

Compared to the female group (β = 0.23, 95% CI: 0.19 to 0.28), the male group exhibited a higher influence of social media usage frequency on the suggestibility of internet health information (β = 0.29, 95% CI: 0.25 to 0.33, *P* < 0.001). Higher effect sizes were also notable in individuals with a smaller BMI (*P* = 0.03), longer sleep latency (*P* < 0.001), and higher SMBG frequency (*P* < 0.001). Individuals with poor GC control (β = 0.36, 95% CI: 0.31 to 0.42) exhibited a stronger correlation compared to those with good control (β = 0.23, 95% CI: 0.20 to 0.26). It is noteworthy that in the layers where interaction analysis results were significant, a positive correlation was consistently observed between social media usage frequency and suggestibility of internet health information.

## Discussion

This study constitutes the inaugural exploration into the association between the utilization frequency of applications and suggestibility by cues from internet health information. The findings proposed herein suggest an enhancement in suggestibility by online information in tandem with the individual’s socialization process. Notably, there exists a U-shaped correlation between the frequency of application use in online glucose management and suggestibility by cues from internet health information. Suggestibility by these cues increases incrementally with the frequency of application usage once a critical threshold is surpassed. Subsequent discussions will address the relationship between application usage frequency and internet health information suggestibility from both macroscopic and microscopic perspectives.

### Demographic characteristics

The propensity for suggestibility appears interlinked with demographic data. Within the cohort examined in this study, males accounted for 38.1%, while females represented 61.9%, with 840 participants aged between 21–40 years (79.1%). This aligns with prior research, which has shown that females are more likely to engage with social media [38]. In the female cohort, a significantly higher proportion demonstrated high suggestibility compared to males (52.3% vs 44.1%, *P* = 0.02). While certain studies suggest no significant disparities in suggestibility between genders [39–41], others advocate that females are more prone to suggestions, corroborating the outcomes of this research [42, 43]. Individuals older than 41 years exhibited the highest percentages of high suggestibility scores (55.49%, *P* = 0.001). Intriguingly, earlier studies have indicated that hypnotic suggestibility scores generally escalate post-40 years of age [43]. Over half of the respondents held a university degree or higher (55.5%), potentially reflecting the younger, more educated demographic prevalent among internet users [16]. Additionally, the study observed that married individuals (*P* < 0.001) and those who frequently communicate with their families (*P* < 0.001) possess heightened suggestibility, suggesting that deeper social connections may enhance suggestibility by suggestions. This analysis posits that women, older individuals, and those with higher educational attainment exhibit stronger suggestibility, indicative of a heightened suggestibility among highly “socialized” groups. Specifically, women display greater social engagement and motivation compared to men [38], and older, more educated individuals typically have richer social and informational exchange experiences than their younger, less educated counterparts. It is worth noting that the average frequency of social media usage among respondents was more than several times per month, likely due to the focus of this study on individuals engaged in online information exchange. This also highlights the pervasive nature of application us [16], a trait that is equally applicable to the online glucose management population. Therefore, from a macroscopic viewpoint, suggestibility escalates with the progression of individual socialization.

### Association between social media usage frequency and suggestibility

This study implemented a multifactorial regression analysis to establish a significant positive correlation between the frequency of application usage and suggestibility, controlling for confounding factors such as gender, age, educational attainment, marital status, living arrangements, frequency of family communication, self-reported health status, BMI (categorized), sleep latency, frequency of self-monitoring of blood glucose (SMBG), and glycemic control status. Previous studies have indicated that individuals prefer interactive information to unidirectional searches, as reflected in the use of applications for receiving and exchanging information [7]. Modern applications offer extensive functionalities, not only allowing users to search for information but also to communicate and even establish connections with strangers. Frequent app users are likely to receive more online information and engage more often in online interactions. The tendency to trust frequently encountered information [44] and data shared by acquaintances and friends [45] may underlie the enhanced suggestibility observed in individuals with higher frequencies.

The correlation between social media usage frequency and suggestibility exhibits a U-shaped pattern. This investigation is the inaugural study to reveal that within the online glucose management population when the social media usage frequency score is below 3.8, each incremental unit in the social media usage frequency score correlates with a reduction of 0.12 in the internet health information suggestibility score (95% CI: -0.19 to -0.05, *P* < 0.001). Conversely, when the social media usage score exceeds 3.8, each additional unit correlates with an increase of 0.46 in the internet health information suggestibility score (95% CI: 0.42 to 0.50, *P* < 0.001). This trend of initial decrease followed by an increase suggests that higher usage frequency leads to exposure to a broader array of information, initially reducing suggestibility due to information diversity. As social media usage intensifies, internet algorithms customize content based on the user’s browsing history, effectively creating a “filter bubble” [46]. Such a scenario enables the tailored content to better satisfy the specific health information needs of patients [47], thereby facilitating acceptance and internalization. Additionally, social networks formed within apps among individuals with similar interests may further reinforce this filter bubble effect [46]. However, the formation of such bubbles is contingent upon the level of socialization and the efficacy of big data algorithms, necessitating a usage frequency above a specific threshold. Hence, the positive correlation between social media usage frequency and internet health information suggestibility becomes apparent only beyond this established threshold, as evidenced by a score exceeding 3.8 in this study.

Notably, the linkage between social media usage frequency and suggestibility by internet health information cues is more pronounced among individuals with higher BMI (*P* = 0.03), longer sleep latency (*P* = 0.001), and increased SMBG frequency (*P* = 0.001). Associations between higher BMI and longer sleep latency with anxiety and stress [48, 49], suggest that individuals with these characteristics may have compromised health states. Such individuals are likely to rely more on internet health information [50], reflecting a heightened dependence on the internet for health-related data. Patients with frequent SMBG are generally more attentive to their health condition, leading to increased interaction and information-seeking behavior through various platforms [51]. Therefore, the suggestibility of those who are more concerned about their health status and experiencing higher levels of stress appears to be more influenced by social media usage frequency.

Collectively, whether examining changes in suggestibility from a broad socio-demographic perspective or investigating user behavioral patterns from a more focused, individual viewpoint—despite observable differences in outcomes—both approaches indicate that suggestibility generally escalates with deeper social engagement.

In regions where primary healthcare resources are limited, diabetic patients may lack adequate interaction with healthcare providers, positioning internet health information as a critical intervention channel for public health authorities. This study suggests that social media usage frequency could serve as a monitoring indicator for governmental agencies. A high social media usage frequency may reflect a population’s overall suggestibility and susceptibility to being influenced by online information. The results of this article not only aid in understanding the online behaviors and psychological traits of online glucose management communities but also inspire regulating the internet health information environment and leveraging the internet for public health education.

### Limitations

This exploratory cross-sectional study provides novel insights into the relationship between app usage frequency and suggestibility to online health information among individuals managing glucose online. However, the study design precludes establishing clear causal relationships and may include unaccounted confounding variables due to the lack of balanced group allocation. The data were exclusively derived from an online survey and relied on self-reported measures, which could be influenced by participants’ internet proficiency. Additionally, the mechanisms through which app usage frequency affects suggestibility were not explored, leaving room for future research. While the suggestibility scale used in this study was newly developed based on social media literature and real-world observations, and underwent preliminary validation through expert review and pilot testing, a more comprehensive validation process, such as confirmatory factor analysis and cross-cultural adaptation, is warranted in future studies.

## Conclusions

The findings of this research indicate that suggestibility by internet health information cues increases with the frequency of social media usage once a certain usage threshold is surpassed. Social media usage frequency may be a significant predictor of susceptibility to internet health information cues among online glucose management populations, thereby facilitating the analysis of online behaviors and psychological characteristics of this demographic.

## Data Availability

The raw data can be obtained from the corresponding author upon reasonable request.

## Abbreviations

BMI: Body measurement index
GC: Glycemic control
IHI: Internet health information
SMBG: Self-monitoring of blood glucose
